# Paxlovid use is associated with lower risk of cardiovascular diseases in COVID-19 patients with autoimmune rheumatic diseases: a retrospective cohort study

**DOI:** 10.1186/s12916-024-03331-0

**Published:** 2024-03-13

**Authors:** Weijie Wang, Yu-Hsun Wang, Ching-Hua Huang, Tsung-Hsueh Hsieh, Gema Hernández Ibarburu, James Cheng-Chung Wei

**Affiliations:** 1 Department of Rheumatology, The Second Affiliated Hospital of Zhejiang Chinese Medical University, Hangzhou, China; 2 Institute of Basic Theory for Chinese Medicine, China Academy of Chinese Medical Science, Beijing, China; 3 Department of Medical Research, Chung Shan Medical University Hospital, Taichung, Taiwan; 4 Department of Allergy, Immunology & Rheumatology, Chung Shan Medical University Hospital, Taichung, Taiwan; 5 Institute of Medicine, Chung Shan Medical University, Taichung, Taiwan; 6 Graduate Institute of Integrated Medicine, China Medical University, Taichung, Taiwan; 7 Department of Medical Research, Taichung Veterans General Hospital, Taichung, Taiwan; 8 Department of Pharmacy, Chung Shan Medical University Hospital, Taichung, Taiwan; 9 Senior TriNetX Healthcare Partnership Manager EMEA, TriNetX, Cambridge, USA

**Keywords:** Paxlovid, COVID-19, Autoimmune rheumatic diseases, Cardiovascular outcomes, TriNetX

## Abstract

**Background:**

Paxlovid has been shown to be effective in reducing mortality and hospitalization rates in patients with coronavirus disease 2019 (COVID-19). It is not known whether Paxlovid can reduce the risk of cardiovascular diseases (CVD) in COVID-19-surviving patients with autoimmune rheumatic diseases (AIRDs).

**Methods:**

TriNetX data from the US Collaborative Network were used in this study. A total of 5,671,395 patients with AIRDs were enrolled between January 1, 2010, and December 31, 2021. People diagnosed with COVID-19 were included in the cohort (*n* = 238,142) from January 1, 2022, to December 31, 2022. The Study population was divided into two groups based on Paxlovid use. Propensity score matching was used to generate groups with matched baseline characteristics. The hazard ratios (HRs) and 95% confidence intervals of cardiovascular outcomes, admission rate, mortality rate, and intensive care unit (ICU) admission rate were calculated between Paxlovid and non-Paxlovid groups. Subgroup analyses on sex, age, race, autoimmune diseases group, and sensitivity analyses for Paxlovid use within the first day or within 2–5 days of COVID-19 diagnosis were performed.

**Results:**

Paxlovid use was associated with lower risks of cerebrovascular complications (HR = 0.65 [0.47–0.88]), arrhythmia outcomes (HR = 0.81 [0.68–0.94]), ischemic heart disease, other cardiac disorders (HR = 0.51 [0.35–0.74]) naming heart failure (HR = 0.41 [0.26–0.63]) and deep vein thrombosis (HR = 0.46 [0.24–0.87]) belonging to thrombotic disorders in AIRD patients with COVID-19. Compared with the Non-Paxlovid group, risks of major adverse cardiac events (HR = 0.56 [0.44–0.70]) and any cardiovascular outcome mentioned above (HR = 0.76 [0.66–0.86]) were lower in the Paxlovid group. Moreover, the mortality (HR = 0.21 [0.11–0.40]), admission (HR = 0.68 [0.60–0.76]), and ICU admission rates (HR = 0.52 [0.33–0.80]) were significantly lower in the Paxlovid group than in the non-Paxlovid group. Paxlovid appears to be more effective in male, older, and Black patients with AIRD. The risks of cardiovascular outcomes and severe conditions were reduced significantly with Paxlovid prescribed within the first day of COVID-19 diagnosis.

**Conclusions:**

Paxlovid use is associated with a lower risk of CVDs and severe conditions in COVID-19-surviving patients with AIRD.

**Supplementary Information:**

The online version contains supplementary material available at 10.1186/s12916-024-03331-0.

## Background

The coronavirus disease 2019 (COVID-19) pandemic has led to more than 6 million confirmed deaths and 15 million estimated deaths and brought great challenges to more than 200 countries [1].

Monoclonal antibodies, multiple antivirals, immunomodulatory drugs, adjuvants, and Chinese herbal medicine have been suggested as drugs for COVID-19 [2, 3]. Paxlovid, an oral antiviral treatment, is composed of two compounds: PF-07,321,332, an oral covalent 3CL protease inhibitor of SARS-CoV-2, and ritonavir, an inhibitor of human immunodeficiency virus (HIV)-1 and HIV-2 protease [4]. Paxlovid has been shown to reduce the mortality and hospitalization rates in patients with COVID-19 [5, 6]. A cohort study using the largest database in Israel demonstrated that Paxlovid appears to be more effective in patients with cardiovascular disease (CVD) and immunosuppressed patients, particularly the elderly [6]. Moreover, observational studies also showed that antivirals such as Paxolid and Monupiravir reduced viral shedding time in COVID-19 patients and the latter effect seems stronger with COVID-19 vaccination [7, 8]. There is growing evidence that infection with COVID-19 is associated with the development of autoimmunity phenomena [9, 10]. A recent analysis reported that 90 reports (99 cases) of new-onset rheumatic autoimmune diseases during or after SARS-CoV-2 infection [11]. Both COVID-19 and autoimmune rheumatic diseases (AIRDs) present with various clinical symptoms involving different organs and systems, including the cardiovascular, renal, and neurological systems [12, 13]. COVID-19 infection may also lead to underlying flare of rheumatic disease. As patients with rheumatic diseases generally have an increased risk of infections and complications [14]. It is critical to control COVID-19 infection for AIRD patients at early time. In our previous studies, we have conducted retrospective cohort studies and noted that the risk of incidental CVDs and autoimmune diseases was substantially higher in the COVID-19 survivors [15, 16]. However, no large-scale study has assessed whether Paxlovid can reduce the risk of CVDs and severe conditions in COVID-19-surviving patients with AIRD. Therefore, the present study aimed to provide some evidence for Paxlovid use in COVID-19 patients with AIRD.

## Methods

The study data were retrieved from the US Collaborative Network from 55 global healthcare organizations in the TriNetX Research Network. The largest worldwide COVID-19 dataset is presently housed in the TriNetX database, which is a global clinical research platform that collects real-time medical data. The database contains demographic details, diagnoses, procedures, medication information, laboratory tests, genomics, and healthcare utilization. The available data included in the database has been introduced in detail in our former research [16]. In the present study, the US Collaborative Network in TriNetX was used to build a cohort out of more than 92 million participants.

### Participants

A total of 5,671,395 patients with AIRD were enrolled between January 1, 2010, and December 31, 2021, from 92,985,898 participants in the US Collaborative Network. People aged ≥ 18 years diagnosed with COVID-19, via either a COVID-19 positive test or ICD-10-CM = U07.1, were included in the cohort (*n* = 238,142) from January 1, 2022, and December 31, 2022. In addition, two groups of participants were selected: 16,396 patients who received Paxlovid within 5 days of COVID-19 diagnosis and 200,777 patients who received none of Paxlovid, molnupiravir, or remdesivir after COVID-19 diagnosis. The index date was defined as the date of the first administration of Paxlovid and the diagnosis of COVID-19, respectively. Patients diagnosed with CVD before the index date were excluded. In the Paxlovid group, patients who were treated with molnupiravir or remdesivir were also excluded. After exclusion, there were 8,805 patients in the Paxlovid group and 110,551 patients in the comparison group.

**Fig. 1:**
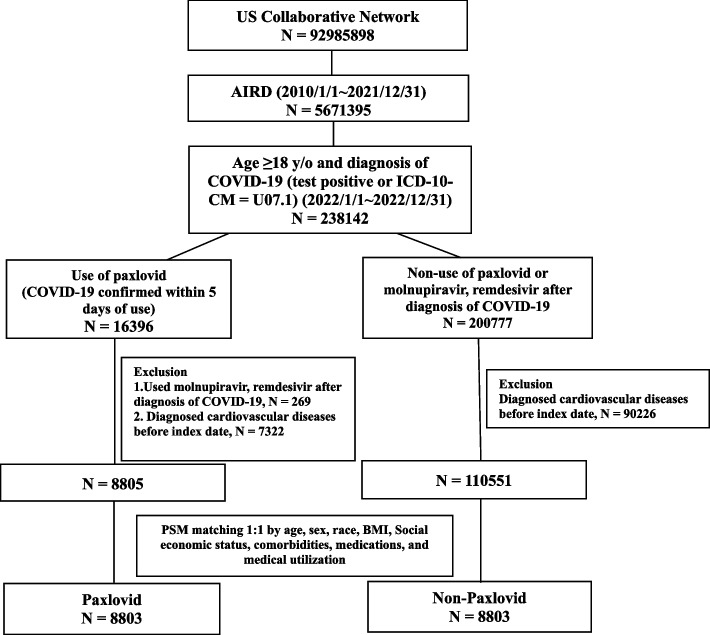
Flow chart of cohort construction

In the study cohort, propensity score matching (PSM) was used to stratify by age, sex, race, body mass index (BMI), socioeconomic status, comorbidities, medications, and medical utilization at a ratio of 1:1. After PSM, 8803 participants in the Paxlovid and 8803 comparisons in the non-Paxlovid groups were selected. Figure 1 shows the flowchart of the cohort.

AIRDs included inflammatory arthritis, connective tissue diseases, autoimmune gastrointestinal diseases, and some endocrine diseases. The following AIRDs were included in the study.

1. Inflammatory arthritis: rheumatoid arthritis [ICD10 = M05 − M06] and ankylosing spondylitis [ICD10 = M45].
2. Connective tissue diseases: vasculitis [ICD10 = M30, M31, L95], atopic dermatitis [ICD10 = L20], psoriatic [ICD10 = L40], systemic lupus erythematosus [ICD10 = M32], dermatomyositis/polymyositis [ICD10 = M33], systemic sclerosis [ICD10 = M34], Sjogren’s syndrome [ICD10 = M35.0,], mixed connective tissue disease [ICD10 = M35.1], Bechet’s disease [ICD10 = M35.2], and polymyalgia rheumatica [ICD10 = M35.3].
3. Autoimmune gastrointestinal diseases: inflammatory bowel disease [ICD10 = K50 − K52], celiac disease [ICD10 = K90.0], and autoimmune hepatitis [ICD10 = K75.4].
4. Endocrine diseases: type 1 diabetes [ICD10 = E10] and autoimmune thyroiditis [ICD10 = E06.3].

In the study cohort, propensity score matching (PSM) was used to stratify by age, sex, race, body mass index (BMI), socioeconomic status (housing/economic circumstances problem [ICD-10-CM = Z59]; problems related to education and literacy [ICD-10-CM = Z55]; employment or unemployment problems [ICD-10-CM = Z56]; occupational exposure to risk factors [ICD-10-CM = Z57]), hypertension [ICD-10-CM = I10]; type 2 diabetes mellitus [ICD-10-CM = E11]; chronic kidney disease [ICD-10-CM = N18]; nicotine dependence [ICD-10-CM = F17]; overweight [ICD-10-CM = E66.3]; alcohol-related disorders [ICD-10-CM = F10], medications (etanercept, adalimumab, golimumab, rituximab, tocilizumab, abatacept, tofacitinib and corticosteroids), and medical utilization (ambulatory, emergency, and inpatient medical treatment) at a ratio of 1:1. These variables were collected within one year before the index date.

### Outcomes

The incidence of CVDs and severe conditions in patients with COVID-19 was assessed from the index date to the end of follow-up (lasting 12 months). The cardiovascular complications and severe conditions in the study were defined as follows in Additional file 1: Table S1.

### Statistical analyses

A built-in Propensity Score Matching (PSM) was employed to create groups with matched baseline characteristics using a greedy nearest neighbor matching approach, with a caliper set at 0.1 pooled standard deviations. The TriNetX was used to match the two groups at a fixed 1:1 ratio by age, sex, race, BMI, socioeconomic status, comorbidities, medications, and medical utilization. Standardized mean differences (SMD) were used to evaluate the balance of baseline characteristics in populations. In General, SMD < 0.1 is considered a small difference. After propensity score matching, a built-in Kaplan–Meier analysis was employed to assess the incidence of outcomes, and the log-rank test was utilized for significance testing. Additionally, a built-in Cox proportional hazard model was applied to estimate the hazard ratios between the Paxlovid and non-Paxlovid groups. The hazard ratio (HR) for cardiovascular outcomes and severe conditions was calculated for both the Paxlovid and Non-Paxlovid groups. Statistical significance was evaluated using the 95% confidence interval (95% CI).

Subgroup analyses were performed to investigate how the risks of cardiovascular outcomes and severe conditions in patients with AIRD differed with respect to sex, age, race, and autoimmune disease groups. In addition, considering possible differences between the early and late use of Paxlovid, a sensitivity analysis was performed for Paxlovid use within the first day and 2 to 5 days of COVID-19 diagnosis.

## Results

### Baseline characteristics of the participants

The demographic characteristics, socioeconomic status, co-morbidities, medications, and medical utilization of the Paxlovid and non-Paxlovid groups before and after PSM are presented in Table 1. The mean age of the participants in the Paxlovid group was about 54 years after matching. Approximately 70.3% of the patients were female and the major race was White (82%). The two groups were well-matched concerning socioeconomic status, comorbidities, medications, and medical utilization (SMD < 0.1).

**Table 1:** Demographic characteristics of Paxlovid and Non-Paxlovid

	Before PSM matching			After PSM matching		
	Paxlovid *N* = 8805	Non-Paxlovid *N* = 110,551	SMD	Paxlovid *N* = 8803	Non-Paxlovid *N* = 8803	SMD
Age, mean ± SD	54.4 ± 15.7	41.9 ± 21.9	0.657	54.4 ± 15.8	54.3 ± 16.2	0.003
Sex						
Female	6134 (69.7)	74444 (67.3)	0.05	6134 (69.7)	6163 (70.0)	0.007
Male	2668 (30.3)	36096 (32.7)	0.051	2668 (30.3)	2640 (30.0)	0.007
Race						
White	7239 (82.2)	76172 (68.9)	0.314	7237 (82.2)	7261 (82.5)	0.007
Black or African American	653 (7.4)	16,000 (14.5)	0.227	653 (7.4)	645 (7.3)	0.003
Asian characteristic(s)	252 (2.9)	3162 (2.9)	< 0.001	252 (2.9)	248 (2.8)	0.003
BMI	3,266	39,979		3,266	3,175	
< 18.5	101 (1.1)	5681 (5.1)	0.230	101 (1.1)	36 (0.4)	0.084
18.5–24.9	925 (10.5)	11987 (10.8)	0.011	925 (10.5)	846 (9.6)	0.03
25–29.9	1253 (14.2)	12510 (11.3)	0.087	1253 (14.2)	1170 (13.3)	0.027
≥ 30	1512 (17.2)	16056 (14.5)	0.073	1512 (17.2)	1517 (17.2)	0.002
Mean ± SD	29.8 ± 6.8	27.9 ± 7.8	0.258	29.8 ± 6.8	30.0 ± 6.7	0.033
Socioeconomic status						
Housing/economic circumstances problem	27 (0.3)	431 (0.4)	0.014	27 (0.3)	14 (0.2)	0.031
Problems related to education and literacy	12 (0.1)	153 (0.1)	0.001	12 (0.1)	10 (0.1)	0.006
Employment or unemployment problems	12 (0.1)	156 (0.1)	0.001	12 (0.1)	10 (0.1)	0.006
Occupational exposure to risk factors	10 (0.1)	43 (0.0)	0.027	10 (0.1)	10 (0.1)	< 0.001
Comorbidities						
Hypertension	3113 (35.4)	24,605 (22.3)	0.292	3113 (35.4)	3092 (35.1)	0.005
Type 2 diabetes mellitus	1450 (16.5)	12,122 (11.0)	0.16	1450 (16.5)	1428 (16.2)	0.007
Chronic kidney disease	332 (3.8)	4253 (3.8)	0.004	332 (3.8)	287 (3.3)	0.028
Nicotine dependence	368 (4.2)	5261 (4.8)	0.028	368 (4.2)	305 (3.5)	0.037
Overweight	109 (1.2)	1328 (1.2)	0.003	109 (1.2)	76 (0.9)	0.037
Alcohol-related disorders	65 (0.7)	1084 (1.0)	0.026	65 (0.7)	65 (0.7)	< 0.001
Medications						
Etanercept	51 (0.6)	497 (0.4)	0.018	51 (0.6)	50 (0.6)	0.002
Adalimumab	151 (1.7)	1478 (1.3)	0.031	151 (1.7)	125 (1.4)	0.024
Golimumab	21 (0.2)	136 (0.1)	0.027	21 (0.2)	16 (0.2)	0.012
Rituximab	25 (0.3)	500 (0.5)	0.028	25 (0.3)	29 (0.3)	0.008
Tocilizumab	17 (0.2)	159 (0.1)	0.012	17 (0.2)	17 (0.2)	< 0.001
Abatacept	20 (0.2)	227 (0.2)	0.005	20 (0.2)	17 (0.2)	0.007
Tofacitinib	19 (0.2)	313 (0.3)	0.013	19 (0.2)	18 (0.2)	0.002
Corticosteroids	2209 (25.1)	27,193 (24.6)	0.011	2209 (25.1)	2099 (23.8)	0.029
Medical utilization						
Ambulatory	8412 (95.5)	92,022 (83.2)	0.407	8410 (95.5)	8355 (94.9)	0.029
Emergency	1502 (17.1)	24,233 (21.9)	0.123	1502 (17.1)	1370 (15.6)	0.041
Inpatient Encounter	529 (6.0)	10931 (9.9)	0.144	529 (6.0)	527 (6.0)	0.001

### Incidence of cardiovascular complications in the two groups

**Fig. 2:**
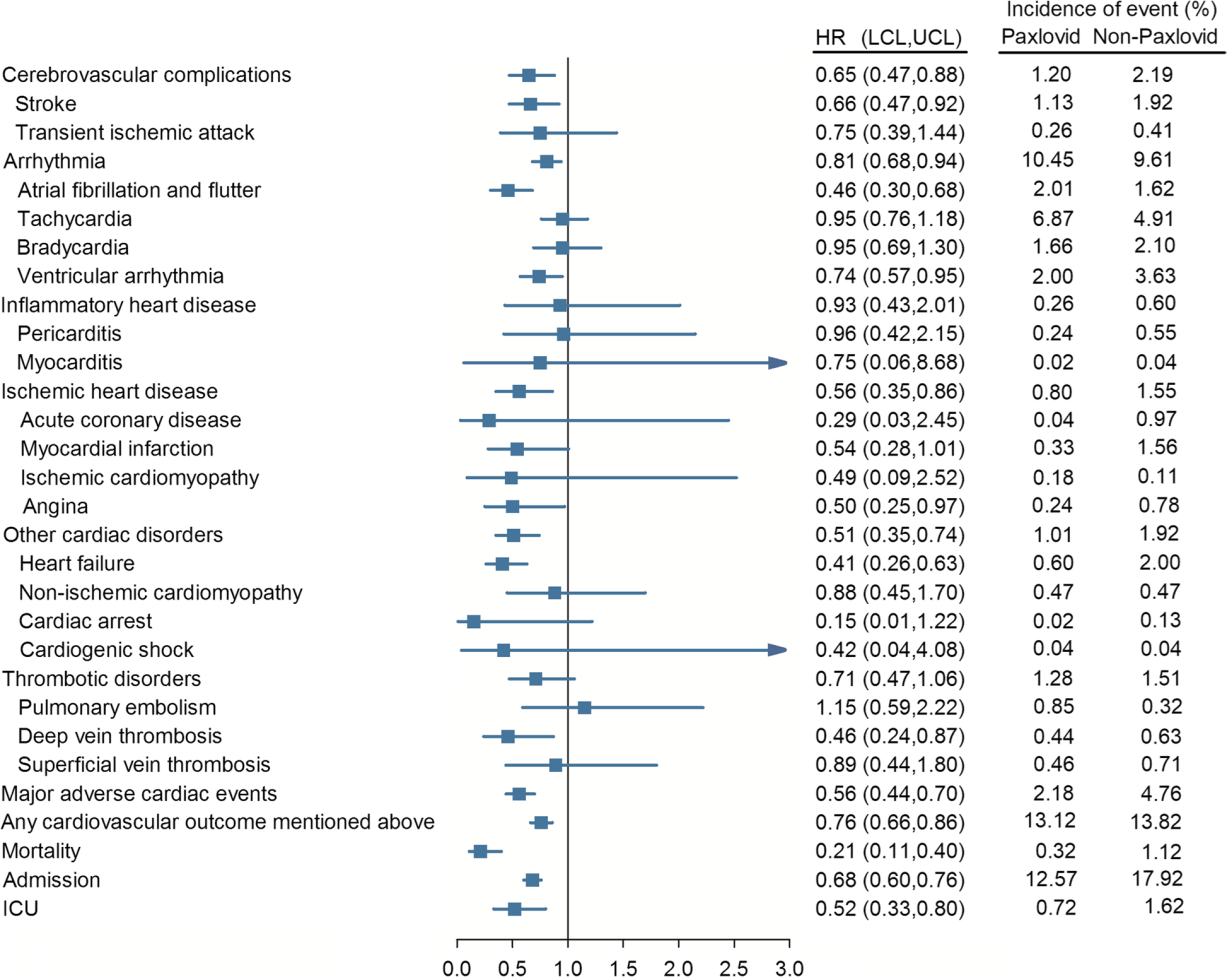
Forest plot of the risk of outcomes among those exposed to Paxlovid compared to non-Paxlovid

The risks of cardiovascular outcomes in the Paxlovid and non-Paxlovid groups were assessed (Fig. 2 and Additional file 1: Table S2). The 12-month follow-up of the patients showed that Paxlovid reduced the risk of CVDs and severe conditions in COVID-19-surviving patients with AIRD.

Paxlovid was associated with lower risks of cerebrovascular complications (HR [95% CI] = 0.65 [0.47–0.88]) such as stroke (HR = 0.66 [0.47–0.92]). Moreover, the risks of arrhythmia outcomes (HR = 0.81 [0.68–0.94]) such as atrial fibrillation and flutter (HR = 0.46 [0.30–0.68]) and ventricular arrhythmia (HR = 0.74 [0.57–0.95]) were reduced. Ischemic heart disease (HR = 0.56 [0.35–0.86]) such as angina (HR = 0.50 [0.25–0.97]) also exhibited lower risks in the Paxlovid group. There were decreased risks of other cardiac disorders (HR = 0.51 [0.35–0.74]) naming heart failure (HR = 0.41 [0.26–0.63]). Deep vein thrombosis (HR = 0.46 [0.24–0.87]) belonging to thrombotic disorders also exhibited significantly lower risks in the Paxlovid group.

Compared with the Non-Paxlovid group, there were decreased risks of MACE (major adverse cardiac events) (HR = 0.56 [0.44–0.70]) and any of the above-mentioned cardiovascular outcomes (HR = 0.76 [0.66–0.86]).

Moreover, the mortality rate in the Paxlovid group was significantly lower than that in the non-Paxlovid group (HR = 0.21 [0.11–0.40]). Finally, the use of Paxlovid reduced the admission (HR = 0.68 [0.60–0.76]) and the ICU admission rates (HR = 0.52 [0.33–0.80]) in the Paxlovid group.

**Fig. 3:**
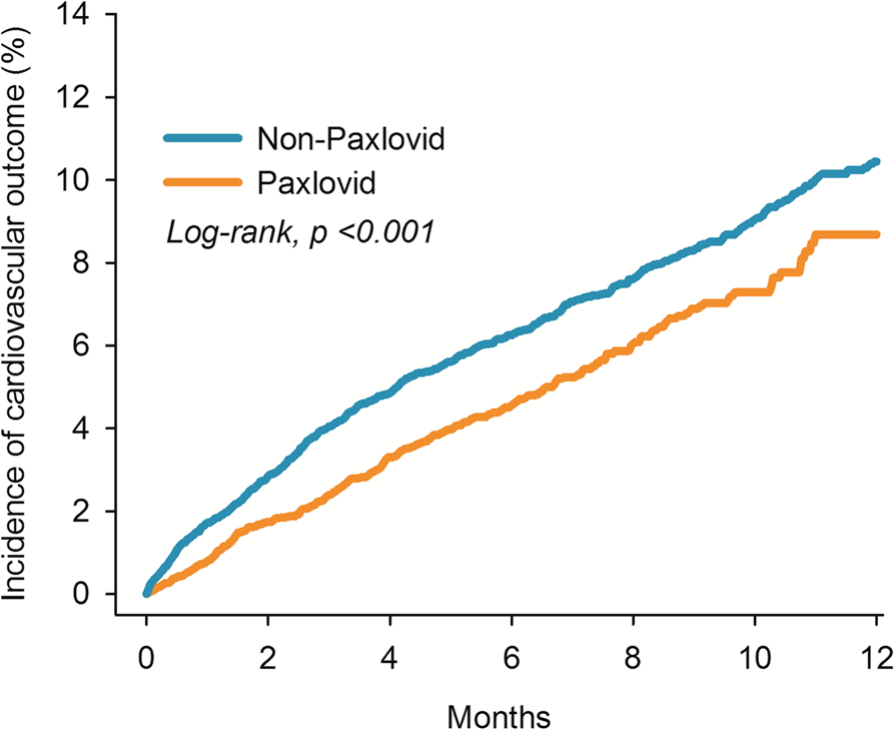
Kaplan–Meier plot for risk of outcomes

The Kaplan–Meier curve of incidence of the cardiovascular outcomes also indicated a difference of probability between the two groups in Fig. 3 (*Log-rank* test, *P* < 0.001).

### Subgroup analyses

**Fig. 4:**
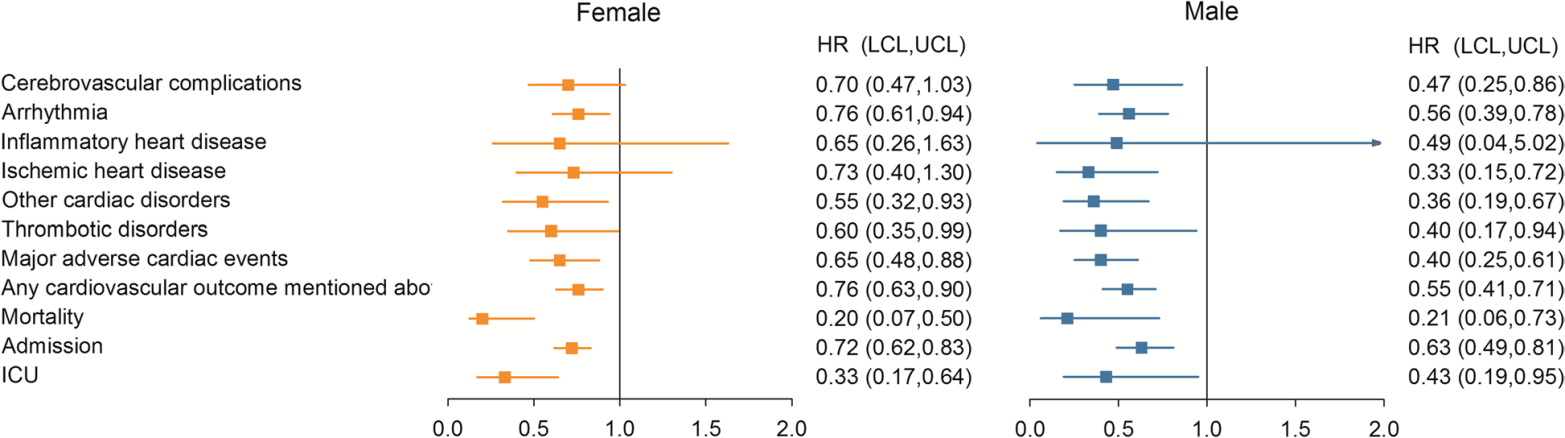
Forest plot of the risk of outcomes for stratification of sex

The risks of CVDs in subgroups were evaluated based on sex, age, and race. Both male and female patients in the Paxlovid group exhibited a significant reduction in the risks of MACE and any cardiovascular outcome, compared with those in the non-Paxlovid group. Compared to the female group, Paxlovid seems to have had a stronger effect in the male group with lower risks of MACE and any cardiovascular outcome. Moreover, the Paxlovid group had significantly lower risks of mortality, admission, and ICU rates in both male and female subgroups. The female subgroup in the Paxlovid group had significantly lower risks of arrhythmia (HR = 0.76 [0.61–0.94]), thrombotic disorders (HR = 0.60[0.35–0.99]), and other cardiac disorders (HR = 0.55 [0.32–0.93]). By contrast, the male subgroup in the Paxlovid group had lower risks of cerebrovascular complications (HR = 0.47 [0.25–0.86]), arrhythmia (HR = 0.56 [0.39–0.78]), THD (HR = 0.33 [0.15–0.72]), other cardiac disorders (HR = 0.36 [0.19–0.67]) and thrombotic disorders (HR = 0.40 [0.17–0.94]) than the male subjects in the Non-Paxlovid group (Additional file 1: Table S3 and Fig. 4).

The middle-aged (aged 45–64 years) and elderly (aged ≥ 65 years) subgroups in the Paxlovid group had a significantly reduced risk of MACE and any cardiovascular outcome. Moreover, the middle-aged subgroups in the Paxlovid group exhibited significantly lower risks of arrhythmia (HR = 0.61 [0.43–0.86]) and thrombotic disorders (HR = 0.44 [0.20–0.94]). The elderly group in the Paxlovid group had lower risks of cerebrovascular complications (HR = 0.55 [0.35–0.85]), arrhythmia (HR = 0.68 [0.50–0.90]), and other cardiac disorders (HR = 0.34 [0.18–0.62]) than the elderly in the non-Paxlovid group. The risk of mortality rate was lower in the middle-aged and elderly subgroups of the Paxlovid group. In addition, the younger (aged 20–44 years) and elderly patients in the Paxlovid group had a significantly reduced risk of admission rate.

**Fig. 5:**
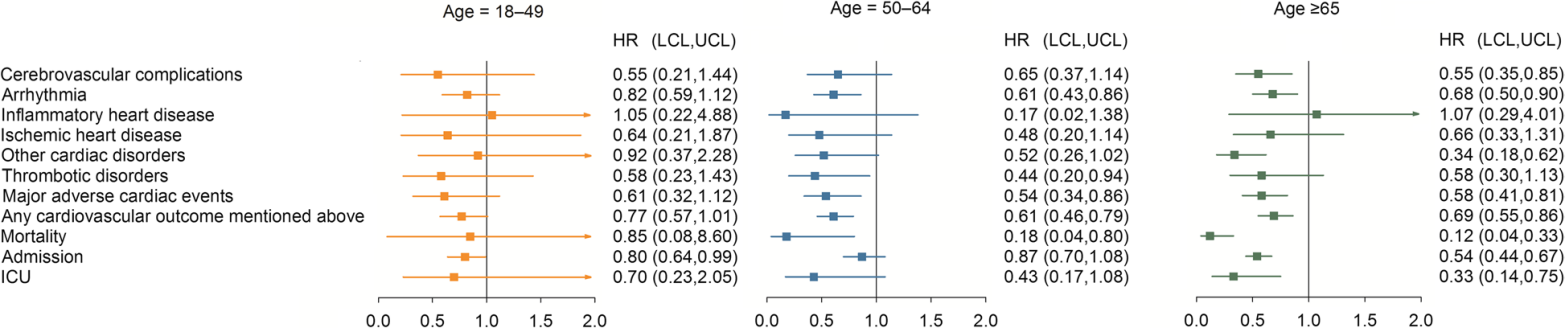
Forest plot of the risk of outcomes for stratification of age

Finally, the elderly patients in the Paxlovid group had a significantly lower risk of ICU rate (HR = 0.33 [0.14–0.75]) than those in the Non-Paxlovid group (Additional file 1: Table S4 and Fig. 5).

With respect to race, the Black group in the Paxlovid group had significantly lower risks of MACE (HR = 0.07 [0.01–0.54]) and any cardiovascular outcome (HR = 0.32 [0.10–0.61]) than that in the non-Paxlovid group. Moreover, the Black subgroup in the Paxlovid group had significantly lower risks of arrhythmia (HR = 0.38 [0.18–0.81]) and thrombotic disorders (HR = 0.13 [0.01–1.05]). The Asian subgroup in the Paxlovid group had a significantly lower risk of arrhythmia (HR = 0.29 [0.03–2.68]) than that in the non-Paxlovid group. The risk of admission rate was significantly reduced in the White and Asian subgroups of the Paxlovid group. In addition, the Asian subgroup in the Paxlovid group had a significantly lower risk of mortality rate (HR = 0.51 [0.05–5.58]) than that in the non-Paxlovid group (Additional file 1: Table S5).

The risks of CVDs on different autoimmune diseases were evaluated in Additional file 1:Tables S6 and S7. The ankylosing spondylitis subgroup in the Paxlovid group had a significantly reduced risk of MACE (HR = 0.28 [0.13–0.59]) and any cardiovascular outcome (HR = 0.69 [0.48–0.99]). Moreover, the same subgroup in the Paxlovid group had significantly lower risks of cerebrovascular complications (HR = 0.30 [0.11–0.80]), IHD (HR = 0.11 [0.02–0.87]) and other cardiac disorders (HR = 0.17 [0.05–0.55]) than in the non-Paxlovid group. The psoriasis subgroup in the Paxlovid group had a significantly reduced risk of any cardiovascular outcome (HR = 0.65 [0.40–0.98]) whereas rheumatoid arthritis and systemic lupus erythematosus subgroups in the Paxlovid group had a significantly lower risk of admission rate (HR = 0.64 [0.44–0.90] and HR = 0.46 [0.23–0.90]).

### Sensitivity analyses

Paxlovid is prescribed for five consecutive days to patients with mild to moderate COVID-19 disease. Therefore, Paxlovid use within the first day and within 2–5 days of diagnosis was next assessed to evaluate whether the patients received the medication on time. The risks of cardiovascular outcomes were reduced significantly when Paxlovid was used within the first day of COVID-19 diagnosis. In addition, the mortality (HR = 0.20 [0.09–0.45]), admission (HR = 0.70 [0.61–0.79]), and ICU admission rates (HR = 0.41 [0.23–0.71]) were also reduced significantly when Paxlovid was used within the first day. However, when Paxlovid was used within days 2–5 days of COVID-19 diagnosis, only arrhythmia (HR = 0.46 [0.23–0.91]), MACE (HR = 0.34 [0.13–0.85]) and any cardiovascular outcome (HR = 0.44 [0.26–0.74]) had lower risks compared with the non-Paxlovid group (Table 2). Moreover, the risks of the outcomes from the second day to the fifth day were also evaluated (Additional file 1: Tables S8 and S9). Since the sample for each day was rather small, the risks of the outcomes on each day were not significantly reduced.

**Table 2:** Risk of outcomes exposed to Paxlovid compared to non-Paxlovid

	Paxlovid		Non-Paxlovid		
	*N*	No. of event	*N*	No. of event	HR
**Use Paxlovid within the first day of a diagnosed COVID-19 infection**					
Cerebrovascular complications	7190	49	7190	117	**0.57 (0.40–0.79)**
Arrhythmia	7190	172	7190	321	**0.73 (0.60–0.87)**
Inflammatory heart disease	7190	10	7190	23	**0.41 (0.17–0.97)**
Ischemic heart disease	7190	24	7190	57	**0.61 (0.37–0.99)**
Other cardiac disorders	7190	30	7190	77	**0.51 (0.33–0.77)**
Thrombotic disorders	7190	26	7190	73	**0.48 (0.30–0.75)**
Major adverse cardiac events	7190	84	7190	197	**0.60 (0.46–0.77)**
Any cardiovascular outcome mentioned above	7190	261	7190	519	**0.67 (0.57–0.78)**
Mortality	7190	10	7190	46	**0.20 (0.09–0.45)**
Admission	7190	369	7190	690	**0.70 (0.61–0.79)**
ICU	7190	17	7190	58	**0.41 (0.23–0.71)**
**Use Paxlovid within days 2 to 5 of a diagnosed COVID-19 infection**					
Cerebrovascular complications	407	10	407	12	0.44 (0.15–1.24)
Arrhythmia	407	12	407	28	**0.46 (0.23–0.91)**
Inflammatory heart disease	407	10	407	10	0.66 (0.05–7.57)
Ischemic heart disease	407	10	407	10	0.36 (0.07–1.72)
Other cardiac disorders	407	10	407	10	0.42 (0.11–1.57)
Thrombotic disorders	407	10	407	10	0.46 (0.11–1.78)
Major adverse cardiac events	407	10	407	19	**0.34 (0.13–0.85)**
Any cardiovascular outcome mentioned above	407	20	407	48	**0.44 (0.26–0.74)**
Mortality	407	10	407	10	0.15 (0.01–1.19)
Admission	407	35	407	39	1.00 (0.63–1.58)
ICU	407	10	407	10	0.32 (0.06–1.55)

If the patient’s count is 10 or less, the results indicate a count of 10

*N/A* Not applicable

## Discussion

The present study showed that treatment with Paxlovid, particularly within the first day of COVID-19 diagnosis could significantly reduce the risks of cardiovascular complications including cerebrovascular complications, arrhythmia, IHD, and thromboembolic disorders in COVID-19-surviving patients with AIRD. The risks of MACE and any cardiovascular complications were also reduced after Paxlovid use. Moreover, Paxlovid reduced the mortality, admission, and ICU admission rates in COVID-19-surviving patients with AIRD which was consistent with other studies on usual participants [17–19]. Additionally, we also performed a side-by-side analysis in patients without autoimmune diseases and found that Paxlovid decreased the risk of MACE (HR = 0.75 [0.69–0.81]) and any cardiovascular outcome mentioned above (HR = 0.85 [0.80–0.88]) (Additional file 1: Table S10, S11). Kaplan–Meier analysis demonstrated the same result (log-rank, *P* < 0.001) (Additional file 1: Fig. S1). However, the effect of Paxlovid seemed stronger with more CVDs in patients with autoimmune diseases. A recent real-life study in 35 Chinese patients with SARS-CoV-2 infection also demonstrated that early treatment of Paxlovid with patients who are immunocompromised (including seven with autoimmune rheumatic conditions) got satisfactory results [20]. However, another multicenter randomized controlled study illustrated that Paxlovid showed no significant reduction in the risk of all-cause mortality for severe adult patients with COVID-19 on day 28 which may be owing to a small number of patients recruited [21].

Most of the AIRD patients (25.1% in the Paxlovid group and 23.8% in the non-Paxlovid group) received corticosteroid treatment. It has been reported that Prednisolone was safe to coadminister NMVr. Additionally, Methylprednisone and Prednisone had potential interaction with NMVr requiring dose adjustment or temporary discontinuation of the drug. Therefore, the ani-inflammatory drug interactions with Paxlovid were not as strong as some CVD medications [22]. Sex-specific risk factors have been identified in autoimmune diseases and CVDs [23, 24]. Younger females are usually protected from CVDs compared with age-matched. However, females tend to develop CVDs following menopause [25]. Systemic lupus erythematosus, rheumatoid arthritis, and Sjogren’s syndrome are more common in women than men [26–28]. By contrast, male patients easily suffered from ankylosing spondylitis [29]. In the present study, Paxlovid significantly tended to protect male patients with AIRD from cardiovascular risks compared with women. Moreover, Paxlovid use reduced risks of CVD more evidently in patients with ankylosing spondylitis rather than in female-dominated autoimmune diseases, including Systemic lupus erythematosus, rheumatoid arthritis, and Sjogren’s syndrome. A large amount of evidence indicated that elderly people infected with SARS-CoV-2 experience severe COVID-19 and have a higher mortality than young people [30, 31].In the present study, Paxlovid reduced CVD risks, severe conditions, and mortality rates in the elderly patients(age ≥ 65 years) with AIRD more significantly than the age-matched non-Paxlovid subjects in comparison with younger patients with COVID-19. Overall, these results indicated that Paxlovid could produce stronger therapeutic effects in moderate and severe COVID-19 patients with AIRDs. Mulnupiravir, controversially banned by EMA, may also have a similar efficacy to Paxlovid against cardiovascular events, based upon both animal studies and observational ones [7, 8, 32]. Large cohort studies regarding cardiovascular outcomes may also need to be performed.

Nearly 23% of reported COVID-19 deaths were those of Black people, even though they account for roughly 13% of the US population [33]. Racial disparities in COVID-19 have been observed and documented across geographical regions [34–36]. Many studies have revealed that Black patients experience more severe COVID-19 outcomes than White patients [37–39]. Of note, in the present study, Paxlovid reduced the risks of cardiovascular complications more significantly, particularly of MACE (HR = 0.07 [0.009–0.54]) in Black people than in White and Asian people.

Paxlovid is usually prescribed for five consecutive days to patients with mild or moderate COVID-19. In the present study, most of the patients (*n* = 7190) received Paxlovid within the first day of COVID-19 diagnosis. Moreover, Paxlovid use within the first day of COVID-19 diagnosis could reduce the risks of CVD, mortality, and severe conditions rather than Paxlovid use within days 2–5 of COVID-19 diagnosis. Although the risks of Paxlovid use from the second day to the fifth day were also assessed, the results were not significant owing to the small number of available patients for a particular day. These results suggest that Paxlovid should be recommended as soon as COVID-19 diagnosis is confirmed. Vaccination remains the most cost-effective tool against COVID-19 mortality, especially for high-risk patients, to be preferred to anti-virals considering not only the cost involved, but also the risk of developing drug resistance with widespread use of antivirals [7]. Therefore, vaccination before the onset of COVID-19 and Paxlovid treatment in early time could have great effects to reduce the risks.

Our study has several limitations. First, although autoimmune disease stratification was implemented for the subgroup analysis, the information on the disease activity of the autoimmune diseases could not be retrieved from TriNetX. Secondly, even though the treatment start time of Paxlovid was evaluated, Paxlovid dose data could not be obtained from the database. Due to the limitations of the TrinetX platform, we could not match the same index date between both groups. It would raise the potential for immortal time bias. To validate the robustness of our study, we performed a sensitivity analysis where both group's dates of index are COVID-19(Additional file 1: TableS12). The number of Asian people in our study was rather small, which may have produced a racial bias in the results. Health insurance coverage had a significant impact on the utilization of healthcare services during the COVID-19 pandemic [40]. However, healthcare insurance status could not be obtained from TrinetX which may produce potential confounding.

In addition, PSM was performed to avoid bias, but misclassification bias and residual confounding could not be completely avoided because of certain disadvantages of an electronic health record database. Furthermore, COVID-19 infection itself increases the risk of cardiovascular events, especially in unvaccinated patients. Although the study showed a reduction in cardiovascular events after the use of Paxlovid, it is not clear whether the reduction is due to the drug itself or the indirect effect of effective control of the viral infection. Finally, some immunosuppressants and biologic treatments may have adverse effects on the cardiovascular risks of patients with AIRD and some drugs may have complex drug-drug interactions with Paxlovid [5].

## Conclusions

Taken together, in this retrospective cohort study, Paxlovid use was associated with lower risks of CVDs and severe conditions in COVID-19-surviving patients with AIRD.

### Supplementary Information

**Additional file 1:** **Table S1.** Outcome Definitions of cardiovascular complications and severe conditions. **Table S2.** Risk of outcomes exposed to Paxlovid compared to Non-Paxlovid. **Table S3.** Sex stratification of risk of outcomes exposed to Paxlovid compared to Non-Paxlovid. **Table S4.** Age stratification of risk of outcomes exposed to Paxlovid compared to Non-Paxlovid. **Table S5.** Race stratification of risk of outcomes exposed to Paxlovid compared to non-Paxlovid. **Table S6.** Autoimmune diseases stratification of risk of outcomes exposed to Paxlovid compared to Non-Paxlovid. **Table S7.** Autoimmune diseases stratification of risk of outcomes exposed to Paxlovid compared to Non-Paxlovid. **Table S8.** Risk of outcomes exposed to Paxlovid compared to non-Paxlovid based on different time. **Table S9.** Risk of outcomes exposed to Paxlovid compared to Non-Paxlovid based on different time. **Table S10.** Demographic characteristics of Paxlovid and Non-Paxlovid in non-autoimmune population. **Table S11.** Risk of outcomes exposed to Paxlovid compared to Non-Paxlovid in non-autoimmune population. **Table S12.** Sensitivity analysis for risk of outcomes exposed to Paxlovid compared to Non-Paxlovid indexed to COVID-19 onset. **Table S13.** Sensitivity analysis for risk of outcomes exposed to Paxlovid compared to Non-Paxlovid with a five-day washout period. **Fig.S1.** Kaplan-Meier plot for risk of cardiovascular diseases in non-autoimmune population.

**Additional file 2:** **Table S1.** STROBE Statement—Checklist of items that should be included in reports of *cohort studies.*

## Data Availability

The data that support the findings of this study are available from the TriNetX Analytics Network. https://trinetx.com.

## Abbreviations

AIRD: Autoimmune rheumatic disease
BMI: Body mass index
COVID-19: Coronavirus disease 2019
CVD: Cardiovascular disease
HIV: Human immunodeficiency virus
HR: Hazard ratios
ICU: Intensive care unit
PSM: Propensity score matching
SMD: Standardized mean differences
