# Supplementary material for: Paxlovid use is associated with lower risk of cardiovascular diseases in COVID-19 patients with autoimmune rheumatic diseases: a retrospective cohort study
Source: BMC Med. 2024 Mar 13;22:117. doi: 10.1186/s12916-024-03331-0 (PMC10938827; doi:10.1186/s12916-024-03331-0)
Supplement: Supplementary file 1 — Additional file 1: Table S1. Outcome Definitions of cardiovascular complications and severe conditions. Table S2. Risk of outcomes exposed to Paxlovid compared to Non-Paxlovid. Table S3. Sex stratification of risk of outcomes exposed to Paxlovid compared to Non-Paxlovid. Table S4. Age stratification of risk of outcomes exposed to Paxlovid compared to Non-Paxlovid. Table S5. Race stratification of risk of outcomes exposed to Paxlovid compared to non-Paxlovid. Table S6. Autoimmune diseases stratification of risk of outcomes exposed to Paxlovid compared to Non-Paxlovid. Table S7. Autoimmune diseases stratification of risk of outcomes exposed to Paxlovid compared to Non-Paxlovid. Table S8. Risk of outcomes exposed to Paxlovid compared to non-Paxlovid based on different time. Table S9. Risk of outcomes exposed to Paxlovid compared to Non-Paxlovid based on different time. Table S10. Demographic characteristics of Paxlovid and Non-Paxlovid in non-autoimmune population. Table S11. Risk of outcomes exposed to Paxlovid compared to Non-Paxlovid in non-autoimmune population. Table S12. Sensitivity analysis for risk of outcomes exposed to Paxlovid compared to Non-Paxlovid indexed to COVID-19 onset. Table S13. Sensitivity analysis for risk of outcomes exposed to Paxlovid compared to Non-Paxlovid with a five-day washout period. Fig.S1. Kaplan-Meier plot for risk of cardiovascular diseases in non-autoimmune population. [file 12916_2024_3331_MOESM1_ESM.docx]

**Table S1** Outcome Definitions of cardiovascular complications and severe conditions

| No | Disease | Code (ICD10 or CPT codes) |
| --- | --- | --- |
| 1 | **Cerebrovascular complications**:  Stroke  Transient ischemic attack (TIA) | I60−I69  G45 |
| 2 | **Arrhythmia**:  Atrial fibrillation and flutter  Tachycardia  Bradycardia  Ventricular arrhythmia | I48  R00.0, I47  R00.1, I49.8, I49.5  I49 |
| 3 | **Inflammatory heart disease**:  Pericarditis  Myocarditis | I30, I31, I32  I40, I41, I51.4 |
| 4 | **Ischemic heart disease**:  Acute coronary disease  Myocardial infarction  Ischemic cardiomyopathy  Angina | I24  I21, I22  I25.5  I20 |
| 5 | **Other cardiac disorders:**  Heart failure  Non-ischemic cardiomyopathy  Cardiac arrest  Cardiogenic shock | I50  I42  I46  R57.0 |
| 6 | **Thrombotic disorders:**  Pulmonary embolism  Deep vein thrombosis  Superficial vein thrombosis | I26  I80.1, I80.2, I81, I82.0, I82.2, I82.3, I82.4, I82.5 I80.0, I80.3, I80.8, I80.9, I82.1, I82.6, I82.7, I82.8, I82.9 |
| 7 | **Major adverse cardiac events (MACEs):**  Infarction  Ischemic stroke  Hemorrhagic stroke  Heart failure  Ventricular arrhythmia  Cardiac arrest | I21−I22  I63, I65, I66, I67.89  I61−I62  I50  I47.0, I47.2, I49.3, I49.0  I46 |
| 8 | The composite of any cardiovascular outcome was defined as the first incident of any cardiovascular complication investigated in this study |  |
| 9 | **Mortality** | Deceased |
| 10 | **Admission** | Visit: Inpatient Encounter or  Hospital Inpatient Services (UMLS: CPT:1013659); or  Visit: Inpatient Non-acute |
| 11 | **ICU** | Critical Care Services (UMLS: CPT:1013729) |

**Table S2**. Risk of outcomes exposed to Paxlovid compared to Non-paxlovid

|  | No. of event | |  |
| --- | --- | --- | --- |
|  | Paxlovid N = 8803 | Non-Paxlovid N = 8803 | HR |
| **Cerebrovascular complications** | 63 | 123 | **0.65 (0.47–0.88)** |
| Stroke | 56 | 108 | **0.66 (0.47–0.92)** |
| Transient ischemic attack (TIA) | 15 | 24 | 0.75 (0.39–1.44) |
| **Arrhythmia** | 255 | 425 | **0.81 (0.68–0.94)** |
| Atrial fibrillation and flutter | 33 | 90 | **0.46 (0.30–0.68)** |
| Tachycardia | 140 | 200 | 0.95 (0.76–1.18) |
| Bradycardia | 69 | 100 | 0.95 (0.69–1.30) |
| Ventricular arrhythmia | 95 | 174 | **0.74 (0.57–0.95)** |
| **Inflammatory heart disease** | 11 | 18 | 0.93 (0.43–2.01) |
| Pericarditis | 10 | 16 | 0.96 (0.42–2.15) |
| Myocarditis | 10 | 10 | 0.75 (0.06–8.68) |
| **Ischemic heart disease** | 29 | 72 | **0.56 (0.35–0.86)** |
| Acute coronary disease | 10 | 10 | 0.29 (0.03–2.45) |
| Myocardial infarction | 14 | 37 | 0.54 (0.28–1.01) |
| Ischemic cardiomyopathy | 10 | 10 | 0.49 (0.09–2.52) |
| Angina | 12 | 33 | **0.50 (0.25–0.97)** |
| **Other cardiac disorders** | 40 | 101 | **0.51 (0.35–0.74)** |
| Heart failure | 28 | 88 | **0.41 (0.26–0.63)** |
| Non-ischemic cardiomyopathy | 15 | 24 | 0.88 (0.45–1.70) |
| Cardiac arrest | 10 | 10 | 0.15 (0.01–1.22) |
| Cardiogenic shock | 10 | 10 | 0.42 (0.04–4.08) |
| **Thrombotic disorders** | 37 | 72 | 0.71 (0.47–1.06) |
| Pulmonary embolism | 17 | 20 | 1.15 (0.59–2.22) |
| Deep vein thrombosis | 13 | 36 | **0.46 (0.24–0.87)** |
| Superficial vein thrombosis | 13 | 24 | 0.89 (0.44–1.80) |
| **Major adverse cardiac events** | 104 | 239 | **0.56 (0.44–0.70)** |
| **Any cardiovascular outcome mentioned above** | 375 | 648 | **0.76 (0.66–0.86)** |
| **Mortality** | 11 | 66 | **0.21 (0.11–0.40)** |
| **Admission** | 480 | 879 | **0.68 (0.60–0.76)** |
| **ICU** | 29 | 78 | **0.52 (0.33–0.80)** |

If the patient's count is 10 or less, the results indicate a count of 10.

**Table S3** Sex stratification of risk of outcomes exposed to Paxlovid compared to Non-Paxlovid

|  | Paxlovid | | Non-Paxlovid | |  |
| --- | --- | --- | --- | --- | --- |
|  | N | No. of event | N | No. of event | HR |
| **Female** |  |  |  |  |  |
| Cerebrovascular complications | 5,271 | 40 | 5,271 | 79 | 0.70 (0.47–1.03) |
| Arrhythmia | 5,271 | 137 | 5,271 | 249 | **0.76 (0.61–0.94)** |
| Inflammatory heart disease | 5,271 | 10 | 5,271 | 14 | 0.65 (0.26–1.63) |
| Ischemic heart disease | 5,271 | 18 | 5,271 | 35 | 0.73 (0.40–1.30) |
| Other cardiac disorders | 5,271 | 20 | 5,271 | 47 | **0.55 (0.32–0.93)** |
| Thrombotic disorders | 5,271 | 22 | 5,271 | 50 | **0.60 (0.35–0.99)** |
| Major adverse cardiac events | 5,271 | 63 | 5,271 | 131 | **0.65 (0.48–0.88)** |
| Any cardiovascular outcome mentioned above | 5,271 | 207 | 5,271 | 373 | **0.76 (0.63–0.90)** |
| Mortality | 5,271 | 10 | 5,271 | 32 | **0.20 (0.07–0.50)** |
| Admission | 5,271 | 307 | 5,271 | 542 | **0.72 (0.62–0.83)** |
| ICU | 5,271 | 11 | 5,271 | 46 | **0.33 (0.17–0.64)** |
| **Male** |  |  |  |  |  |
| Cerebrovascular complications | 2,332 | 14 | 2,332 | 40 | **0.47 (0.25–0.86)** |
| Arrhythmia | 2,332 | 49 | 2,332 | 116 | **0.56 (0.39–0.78)** |
| Inflammatory heart disease | 2,332 | 10 | 2,332 | 10 | 0.49 (0.04–5.02) |
| Ischemic heart disease | 2,332 | 10 | 2,332 | 33 | **0.33 (0.15–0.72)** |
| Other cardiac disorders | 2,332 | 13 | 2,332 | 47 | **0.36 (0.19–0.67)** |
| Thrombotic disorders | 2,332 | 10 | 2,332 | 22 | **0.40 (0.17–0.94)** |
| Major adverse cardiac events | 2,332 | 27 | 2,332 | 92 | **0.40 (0.25–0.61)** |
| Any cardiovascular outcome mentioned above | 2,332 | 76 | 2,332 | 185 | **0.55 (0.41–0.71)** |
| Mortality | 2,332 | 10 | 2,332 | 19 | **0.21 (0.06–0.73)** |
| Admission | 2,332 | 98 | 2,332 | 211 | **0.63 (0.49–0.81)** |
| ICU | 2,332 | 10 | 2,332 | 27 | **0.43 (0.19–0.95)** |

If the patient's count is 10 or less, the results indicate a count of 10.

**Table S4** Age stratification of risk of outcomes exposed to Paxlovid compared to Non-Paxlovid

|  | Paxlovid | | Non-Paxlovid | |  |
| --- | --- | --- | --- | --- | --- |
|  | N | No. of event | N | No. of event | HR |
| **Age 18-49** |  |  |  |  |  |
| Cerebrovascular complications | 2,722 | 10 | 2,722 | 15 | 0.55 (0.21–1.44) |
| Arrhythmia | 2,722 | 64 | 2,722 | 112 | 0.82 (0.59–1.12) |
| Inflammatory heart disease | 2,722 | 10 | 2,722 | 10 | 1.05 (0.22–4.88) |
| Ischemic heart disease | 2,722 | 10 | 2,722 | 11 | 0.64 (0.21–1.87) |
| Other cardiac disorders | 2,722 | 10 | 2,722 | 12 | 0.92 (0.37–2.28) |
| Thrombotic disorders | 2,722 | 10 | 2,722 | 15 | 0.58 (0.23–1.43) |
| Major adverse cardiac events | 2,722 | 15 | 2,722 | 34 | 0.61 (0.32–1.12) |
| Any cardiovascular outcome mentioned above | 2,722 | 79 | 2,722 | 144 | 0.77 (0.57–1.01) |
| Mortality | 2,722 | 10 | 2,722 | 10 | 0.85 (0.08–8.60) |
| Admission | 2,722 | 136 | 2,722 | 235 | **0.80 (0.64–0.99)** |
| ICU | 2,722 | 10 | 2,722 | 11 | 0.70 (0.23–2.05) |
| **Age 50-64** |  |  |  |  |  |
| Cerebrovascular complications | 2,577 | 19 | 2,577 | 40 | 0.65 (0.37–1.14) |
| Arrhythmia | 2,577 | 49 | 2,577 | 105 | **0.61 (0.43–0.86)** |
| Inflammatory heart disease | 2,577 | 10 | 2,577 | 10 | 0.17 (0.02–1.38) |
| Ischemic heart disease | 2,577 | 10 | 2,577 | 22 | 0.48 (0.20–1.14) |
| Other cardiac disorders | 2,577 | 12 | 2,577 | 31 | 0.52 (0.26–1.02) |
| Thrombotic disorders | 2,577 | 10 | 2,577 | 30 | **0.44 (0.20–0.94)** |
| Major adverse cardiac events | 2,577 | 25 | 2,577 | 65 | **0.54 (0.34–0.86)** |
| Any cardiovascular outcome mentioned above | 2,577 | 80 | 2,577 | 176 | **0.61 (0.46–0.79)** |
| Mortality | 2,577 | 10 | 2,577 | 16 | **0.18 (0.04–0.80)** |
| Admission | 2,577 | 146 | 2,577 | 219 | 0.87 (0.70–1.08) |
| ICU | 2,577 | 10 | 2,577 | 22 | 0.43 (0.17–1.08) |
| **Age ≥65** |  |  |  |  |  |
| Cerebrovascular complications | 2,231 | 29 | 2,231 | 67 | **0.55 (0.35–0.85)** |
| Arrhythmia | 2,231 | 69 | 2,231 | 126 | **0.68 (0.50–0.90)** |
| Inflammatory heart disease | 2,231 | 10 | 2,231 | 10 | 1.07 (0.29–4.01) |
| Ischemic heart disease | 2,231 | 13 | 2,231 | 23 | 0.66 (0.33–1.31) |
| Other cardiac disorders | 2,231 | 13 | 2,231 | 49 | **0.34 (0.18–0.62)** |
| Thrombotic disorders | 2,231 | 13 | 2,231 | 29 | 0.58 (0.30–1.13) |
| Major adverse cardiac events | 2,231 | 49 | 2,231 | 108 | **0.58 (0.41–0.81)** |
| Any cardiovascular outcome mentioned above | 2,231 | 119 | 2,231 | 212 | **0.69 (0.55–0.86)** |
| Mortality | 2,231 | 10 | 2,231 | 41 | **0.12 (0.04–0.33)** |
| Admission | 2,231 | 118 | 2,231 | 264 | **0.54 (0.44–0.67)** |
| ICU | 2,231 | 10 | 2,231 | 28 | **0.33 (0.14–0.75)** |

If the patient's count is 10 or less, the results indicate a count of 10.

**Table S5** Race stratification of risk of outcomes exposed to paxlovid compared to non-paxlovid

|  | Paxlovid | | Non-Paxlovid | |  |
| --- | --- | --- | --- | --- | --- |
|  | N | No. of event | N | No. of event | HR |
| **White** |  |  |  |  |  |
| Cerebrovascular complications | 6,367 | 51 | 6,367 | 109 | 0.63 (0.44–0.88) |
| Arrhythmia | 6,367 | 168 | 6,367 | 316 | 0.74 (0.61–0.90) |
| Inflammatory heart disease | 6,367 | 10 | 6,367 | 19 | 0.47 (0.18–1.20) |
| Ischemic heart disease | 6,367 | 23 | 6,367 | 46 | 0.72 (0.42–1.19) |
| Other cardiac disorders | 6,367 | 31 | 6,367 | 86 | 0.47 (0.31–0.71) |
| Thrombotic disorders | 6,367 | 27 | 6,367 | 63 | 0.61 (0.38–0.97) |
| Major adverse cardiac events | 6,367 | 85 | 6,367 | 195 | 0.60 (0.46–0.77) |
| Any cardiovascular outcome mentioned above | 6,367 | 256 | 6,367 | 490 | 0.72 (0.61–0.83) |
| Mortality | 6,367 | 10 | 6,367 | 34 | 0.27 (0.11–0.61) |
| Admission | 6,367 | 319 | 6,367 | 589 | **0.73 (0.63–0.84)** |
| ICU | 6,367 | 18 | 6,367 | 39 | 0.71 (0.39–1.25) |
| **Black** |  |  |  |  |  |
| Cerebrovascular complications | 465 | 10 | 465 | 10 | 0.27 (0.05–1.28) |
| Arrhythmia | 465 | 10 | 465 | 31 | **0.38 (0.18–0.81)** |
| Inflammatory heart disease | 465 | 0 | 465 | 10 | N/A |
| Ischemic heart disease | 465 | 10 | 465 | 10 | 0.17 (0.02–1.41) |
| Other cardiac disorders | 465 | 0 | 465 | 10 | N/A |
| Thrombotic disorders | 465 | 10 | 465 | 10 | **0.13 (0.01–1.05)** |
| Major adverse cardiac events | 465 | 10 | 465 | 17 | **0.07 (0.01–0.54)** |
| Any cardiovascular outcome mentioned above | 465 | 12 | 465 | 44 | **0.32 (0.10–0.61)** |
| Mortality | 465 | 0 | 465 | 10 | N/A |
| Admission | 465 | 52 | 465 | 66 | 0.96 (0.66–1.39) |
| ICU | 465 | 0 | 465 | 10 | N/A |
| **Asian** |  |  |  |  |  |
| Cerebrovascular complications | 184 | 0 | 184 | 10 | N/A |
| Arrhythmia | 184 | 10 | 184 | 10 | **0.29 (0.03–2.68)** |
| Inflammatory heart disease | 184 | 0 | 184 | 0 | N/A |
| Ischemic heart disease | 184 | 10 | 184 | 10 | 1.31 (0.17–9.90) |
| Other cardiac disorders | 184 | 0 | 184 | 0 | N/A |
| Thrombotic disorders | 184 | 10 | 184 | 10 | 1.06 (0.07–16.95) |
| Major adverse cardiac events | 184 | 10 | 184 | 10 | 0.85 (0.07–9.83) |
| Any cardiovascular outcome mentioned above | 184 | 10 | 184 | 10 | 0.45 (0.12–1.72) |
| Mortality | 184 | 10 | 184 | 10 | **0.51 (0.05–5.58)** |
| Admission | 184 | 10 | 184 | 17 | **0.41 (0.16–1.05)** |
| ICU | 184 | 10 | 184 | 0 | N/A |

If the patient's count is 10 or less, the results indicate a count of 10. N/A: Not applicable.

**Table S6** Autoimmune diseases stratification of risk of outcomes exposed to Paxlovid compared to Non-Paxlovid

|  | Paxlovid | | Non-Paxlovid | |  |
| --- | --- | --- | --- | --- | --- |
|  | N | No. of event | N | No. of event | HR |
| **Rheumatoid arthritis** |  |  |  |  |  |
| Cerebrovascular complications | 903 | 10 | 903 | 15 | 0.72 (0.31–1.65) |
| Arrhythmia | 903 | 28 | 903 | 54 | 0.77 (0.47–1.23) |
| Inflammatory heart disease | 903 | 10 | 903 | 10 | 2.04 (0.10–38.30) |
| Ischemic heart disease | 903 | 10 | 903 | 10 | 0.70 (0.25–1.94) |
| Other cardiac disorders | 903 | 10 | 903 | 12 | 0.59 (0.21–1.58) |
| Thrombotic disorders | 903 | 10 | 903 | 12 | 0.96 (0.38–2.40) |
| Major adverse cardiac events | 903 | 14 | 903 | 25 | 0.70 (0.35–1.35) |
| Any cardiovascular outcome mentioned above | 903 | 44 | 903 | 80 | 0.77 (0.52–1.13) |
| Mortality | 903 | 10 | 903 | 10 | 0.52 (0.09–2.85) |
| Admission | 903 | 47 | 903 | 96 | **0.64 (0.44–0.90)** |
| ICU | 903 | 10 | 903 | 10 | 0.30 (0.06–1.41) |
| **Systemic lupus erythematosus** |  |  |  |  |  |
| Cerebrovascular complications | 224 | 0 | 224 | 0 | N/A |
| Arrhythmia | 224 | 10 | 224 | 11 | 0.62 (0.21–1.84) |
| Inflammatory heart disease | 224 | 0 | 224 | 10 | N/A |
| Ischemic heart disease | 224 | 10 | 224 | 10 | 0.42 (0.04–4.12) |
| Other cardiac disorders | 224 | 0 | 224 | 10 | N/A |
| Thrombotic disorders | 224 | 10 | 224 | 10 | 0.37 (0.04–3.22) |
| Major adverse cardiac events | 224 | 10 | 224 | 10 | 0.51 (0.095–2.73) |
| Any cardiovascular outcome mentioned above | 224 | 10 | 224 | 15 | 0.66 (0.20–1.67) |
| Mortality | 224 | 0 | 224 | 0 | N/A |
| Admission | 224 | 12 | 224 | 30 | **0.46 (0.23–0.90)** |
| ICU | 224 | 0 | 224 | 10 | N/A |
| **Ankylosing spondylitis** |  |  |  |  |  |
| Cerebrovascular complications | 903 | 10 | 903 | 21 | **0.30 (0.11–0.80)** |
| Arrhythmia | 903 | 35 | 903 | 59 | 0.80 (0.53–1.22) |
| Inflammatory heart disease | 903 | 10 | 903 | 10 | 0.28 (0.03–2.49) |
| Ischemic heart disease | 903 | 10 | 903 | 13 | **0.11 (0.02–0.87)** |
| Other cardiac disorders | 903 | 10 | 903 | 23 | **0.17 (0.05–0.55)** |
| Thrombotic disorders | 903 | 10 | 903 | 12 | 0.67 (0.23–1.95) |
| Major adverse cardiac events | 903 | 10 | 903 | 38 | **0.28 (0.13–0.59)** |
| Any cardiovascular outcome mentioned above | 903 | 44 | 903 | 85 | **0.69 (0.48–0.99)** |
| Mortality | 903 | 10 | 903 | 10 | 0.73 (0.13–4.08) |
| Admission | 903 | 53 | 903 | 96 | 0.77 (0.55–1.08) |
| ICU | 903 | 10 | 903 | 10 | 0.17 (0.02–1.33) |

If the patient's count is 10 or less, the results indicate a count of 10.

N/A: Not applicable.

**Table S7** Autoimmune diseases stratification of risk of outcomes exposed to Paxlovid compared to Non-Paxlovid

|  | Paxlovid | | Non-Paxlovid | |  |
| --- | --- | --- | --- | --- | --- |
|  | N | No. of event | N | No. of event | HR |
| **Sjogren’s syndrome** |  |  |  |  |  |
| Cerebrovascular complications | 332 | 0 | 332 | 10 | N/A |
| Arrhythmia | 332 | 11 | 332 | 17 | 0.87 (0.40–1.89) |
| Inflammatory heart disease | 332 | 0 | 332 | 0 | N/A |
| Ischemic heart disease | 332 | 10 | 332 | 10 | 0.24 (0.02–2.08) |
| Other cardiac disorders | 332 | 10 | 332 | 10 | 3.34 (0.63–17.70) |
| Thrombotic disorders | 332 | 10 | 332 | 10 | 0.35 (0.03–3.35) |
| Major adverse cardiac events | 332 | 10 | 332 | 10 | 0.40 (0.08–1.98) |
| Any cardiovascular outcome mentioned above | 332 | 13 | 332 | 26 | 0.69 (0.34–1.35) |
| Mortality | 332 | 0 | 332 | 10 | N/A |
| Admission | 332 | 19 | 332 | 34 | 0.72 (0.40–1.26) |
| ICU | 332 | 0 | 332 | 10 | N/A |
| **Psoriasis** |  |  |  |  |  |
| Cerebrovascular complications | 986 | 10 | 986 | 20 | 0.53 (0.21–1.26) |
| Arrhythmia | 986 | 23 | 986 | 55 | 0.65 (0.39–1.07) |
| Inflammatory heart disease | 986 | 10 | 986 | 10 | 0.25 (0.02–2.23) |
| Ischemic heart disease | 986 | 10 | 986 | 10 | 0.24 (0.02–2.05) |
| Other cardiac disorders | 986 | 10 | 986 | 10 | 0.63 (0.21–1.85) |
| Thrombotic disorders | 986 | 10 | 986 | 10 | 0.76 (0.22–2.64) |
| Major adverse cardiac events | 986 | 11 | 986 | 23 | 0.67 (0.32–1.39) |
| Any cardiovascular outcome mentioned above | 986 | 34 | 986 | 78 | **0.65 (0.40–0.98)** |
| Mortality | 986 | 10 | 986 | 10 | 0.38 (0.07–1.84) |
| Admission | 986 | 54 | 986 | 97 | 0.81 (0.57–1.13) |
| ICU | 986 | 10 | 986 | 10 | 1.47 (0.31–6.94) |

If the patient's count is 10 or less, the results indicate a count of 10.

N/A: Not applicable.

**Table S8** Risk of outcomes exposed to Paxlovid compared to Non-Paxlovid based on different time

|  | Paxlovid | | Non-Paxlovid | |  |
| --- | --- | --- | --- | --- | --- |
|  | N | No. of event | N | No. of event | HR |
| **Use paxlovid on the second day of a diagnosed COVID-19 infection** | | | | | |
| Cerebrovascular complications | 264 | 10 | 264 | 10 | 1.59 (0.26–9.54) |
| Arrhythmia | 264 | 10 | 264 | 16 | 0.60 (0.25–1.41) |
| Inflammatory heart disease | 264 | 10 | 264 | 10 | 1.01 (0.06–16.10) |
| Ischemic heart disease | 264 | 0 | 264 | 10 | N/A |
| Other cardiac disorders | 264 | 10 | 264 | 10 | 0.14 (0.01–1.15) |
| Thrombotic disorders | 264 | 10 | 264 | 10 | 1.14 (0.07–18.20) |
| Major adverse cardiac events | 264 | 10 | 264 | 12 | 0.34 (0.10–1.05) |
| Any cardiovascular outcome mentioned above | 264 | 13 | 264 | 21 | 0.71 (0.35–1.42) |
| Mortality | 264 | 0 | 264 | 10 | N/A |
| Admission | 264 | 23 | 264 | 28 | 0.98 (0.55–1.71) |
| ICU | 264 | 0 | 264 | 10 | N/A |
| Mechanical Ventilation | 264 | 0 | 264 | 0 | N/A |
| **Use paxlovid on the third day of a diagnosed COVID-19 infection** | | | | | |
| Cerebrovascular complications | 104 | 10 | 104 | 10 | 1.88 (0.17–20.70) |
| Arrhythmia | 104 | 10 | 104 | 10 | 1.15 (0.38–3.41) |
| Inflammatory heart disease | 104 | 0 | 104 | 10 | N/A |
| Ischemic heart disease | 104 | 10 | 104 | 10 | 1.91 (0.17–21.00) |
| Other cardiac disorders | 104 | 10 | 104 | 10 | 1.97 (0.17–21.70) |
| Thrombotic disorders | 104 | 10 | 104 | 10 | 1.95 (0.17–21.50) |
| Major adverse cardiac events | 104 | 10 | 104 | 10 | 1.53 (0.25–9.16) |
| Any cardiovascular outcome mentioned above | 104 | 10 | 104 | 10 | 0.98 (0.40–2.34) |
| Mortality | 104 | 10 | 104 | 10 | 0.97 (0.06–15.40) |
| Admission | 104 | 13 | 104 | 12 | 1.10 (0.50–2.40) |
| ICU | 104 | 10 | 104 | 10 | 0.65 (0.10–3.89) |
| Mechanical Ventilation | 104 | 0 | 104 | 10 | N/A |

If the patient's count is 10 or less, the results indicate a count of 10.

N/A: Not applicable.

**Table S9** Risk of outcomes exposed to Paxlovid compared to Non-Paxlovid based on different time

|  | Paxlovid | | Non-Paxlovid | |  |
| --- | --- | --- | --- | --- | --- |
|  | N | No. of event | N | No. of event | HR |
| **Use paxlovid on the fourth day of a diagnosed COVID-19 infection** | | | | | |
| Cerebrovascular complications | 29 | 0 | 29 | 10 | N/A |
| Arrhythmia | 29 | 10 | 29 | 0 | N/A |
| Inflammatory heart disease | 29 | 0 | 29 | 0 | N/A |
| Ischemic heart disease | 29 | 0 | 29 | 0 | N/A |
| Other cardiac disorders | 29 | 0 | 29 | 0 | N/A |
| Thrombotic disorders | 29 | 0 | 29 | 0 | N/A |
| Major adverse cardiac events | 29 | 0 | 29 | 10 | N/A |
| Any cardiovascular outcome mentioned above | 29 | 10 | 29 | 10 | 1.03 (0.06–16.50) |
| Mortality | 29 | 0 | 29 | 0 | N/A |
| Admission | 29 | 10 | 29 | 10 | 0.37 (0.03–3.52) |
| ICU | 29 | 0 | 29 | 0 | N/A |
| Mechanical Ventilation | 29 | 0 | 29 | 0 | N/A |
| **Use paxlovid on the fifth day of a diagnosed COVID-19 infection** | | | | | |
| Cerebrovascular complications | 42 | 10 | 42 | 10 | 0.77 (0.04–12.30) |
| Arrhythmia | 42 | 0 | 42 | 10 | N/A |
| Inflammatory heart disease | 42 | 0 | 42 | 0 | N/A |
| Ischemic heart disease | 42 | 0 | 42 | 10 | N/A |
| Other cardiac disorders | 42 | 0 | 42 | 10 | N/A |
| Thrombotic disorders | 42 | 0 | 42 | 0 | N/A |
| Major adverse cardiac events | 42 | 10 | 42 | 10 | 0.41 (0.03–4.55) |
| Any cardiovascular outcome mentioned above | 42 | 10 | 42 | 10 | 0.41 (0.03–4.55) |
| Mortality | 42 | 0 | 42 | 10 | N/A |
| Admission | 42 | 10 | 42 | 10 | **0.11 (0.01–0.84)** |
| ICU | 42 | 0 | 42 | 10 | N/A |
| Mechanical Ventilation | 42 | 0 | 42 | 10 | N/A |

If the patient's count is 10 or less, the results indicate a count of 10.

N/A: Not applicable.

**Table S10** Demographic characteristics of Paxlovid and Non-Paxlovid in non-autoimmune population

|  | Before PSM matching | |  |  | After PSM matching | |  |  |
| --- | --- | --- | --- | --- | --- | --- | --- | --- |
|  | Paxlovid N = 43885 | Non-Paxlovid N = 856243 | p | SMD | Paxlovid N = 43882 | Non-Paxlovid N = 43882 | p | SMD |
| Age, Mean ± SD | 54.81 ± 16.37 | 39.71 ± 21.60 | 0.000 | 0.788 | 54.80 ± 16.37 | 54.88 ± 16.57 | 0.518 | 0.004 |
| Sex |  |  |  |  |  |  |  |  |
| Female | 24361 (55.51) | 494982 (57.81) | 0.000 | 0.046 | 24361 (55.52) | 24410 (55.63) | 0.739 | 0.002 |
| Male | 16581 (37.78) | 340516 (39.77) | 0.000 | 0.041 | 16581 (37.79) | 16582 (37.79) | 0.994 | 0.000 |
| Race |  |  |  |  |  |  |  |  |
| White | 29668 (67.60) | 494184 (57.72) | 0.000 | 0.206 | 29668 (67.61) | 29863 (68.05) | 0.159 | 0.010 |
| Black or African American | 5298 (12.07) | 153617 (17.94) | 0.000 | 0.165 | 5298 (12.07) | 5337 (12.16) | 0.687 | 0.003 |
| Asian characteristic(s) | 2033 (4.63) | 43145 (5.04) | 0.000 | 0.019 | 2033 (4.63) | 1932 (4.40) | 0.101 | 0.011 |
| BMI |  |  |  |  |  |  |  |  |
| <18.5 | 117 (0.27) | 20147 (2.35) | 0.000 | 0.184 | 117 (0.27) | 66 (0.15) | 0.000 | 0.025 |
| 18.5-24.9 | 1162 (2.65) | 36409 (4.25) | 0.000 | 0.088 | 1162 (2.65) | 1091 (2.49) | 0.130 | 0.010 |
| 25-29.9 | 1882 (4.29) | 36612 (4.28) | 0.899 | 0.001 | 1882 (4.29) | 1804 (4.11) | 0.189 | 0.009 |
| ≥30 | 2266 (5.16) | 43519 (5.08) | 0.452 | 0.004 | 2266 (5.16) | 2280 (5.20) | 0.831 | 0.001 |
| Mean ± SD | 30.02 ± 6.56 | 27.20 ± 7.75 | 0.000 | 0.392 | 30.02 ± 6.56 | 29.97 ± 6.36 | 0.707 | 0.008 |
| Social economic status |  |  |  |  |  |  |  |  |
| Housing/economic circumstances problem | 109 (0.25) | 1874 (0.22) | 0.198 | 0.006 | 109 (0.25) | 77 (0.18) | 0.019 | 0.016 |
| Problems related to education and literacy | 14 (0.03) | 318 (0.04) | 0.577 | 0.003 | 14 (0.03) | 10 (0.02) | 0.414 | 0.006 |
| Employment or unemployment problems | 43 (0.10) | 863 (0.10) | 0.857 | 0.001 | 43 (0.10) | 31 (0.07) | 0.163 | 0.009 |
| Occupational exposure to risk factors | 23 (0.05) | 558 (0.07) | 0.305 | 0.005 | 23 (0.05) | 16 (0.04) | 0.262 | 0.008 |
| Comorbidities |  |  |  |  |  |  |  |  |
| Hypertension | 12104 (27.58) | 91071 (10.64) | 0.000 | 0.441 | 12101 (27.58) | 12063 (27.49) | 0.774 | 0.002 |
| Type 2 diabetes mellitus | 4500 (10.25) | 36212 (4.23) | 0.000 | 0.234 | 4499 (10.25) | 4453 (10.15) | 0.608 | 0.003 |
| Chronic kidney disease | 1039 (2.37) | 11755 (1.37) | 0.000 | 0.073 | 1039 (2.37) | 875 (1.99) | 0.000 | 0.026 |
| Nicotine dependence | 1324 (3.02) | 20458 (2.39) | 0.000 | 0.039 | 1324 (3.02) | 1232 (2.81) | 0.065 | 0.012 |
| Overweight | 497 (1.13) | 5856 (0.68) | 0.000 | 0.047 | 497 (1.13) | 420 (0.96) | 0.011 | 0.017 |
| Alcohol related disorders | 303 (0.69) | 5070 (0.59) | 0.009 | 0.012 | 303 (0.69) | 268 (0.61) | 0.142 | 0.010 |
| Medications |  |  |  |  |  |  |  |  |
| Etanercept | 10 (0.02) | 29 (0.00) | 0.000 | 0.017 | 10 (0.02) | 10 (0.02) | 1.000 | 0.000 |
| Adalimumab | 26 (0.06) | 209 (0.02) | 0.000 | 0.017 | 26 (0.06) | 23 (0.05) | 0.668 | 0.003 |
| Golimumab | 10 (0.02) | 10 (0.00) | 0.000 | 0.020 | 10 (0.02) | 0 (0.00) | 0.002 | 0.021 |
| Rituximab | 28 (0.06) | 618 (0.07) | 0.523 | 0.003 | 28 (0.06) | 20 (0.05) | 0.248 | 0.008 |
| Tocilizumab | 10 (0.02) | 157 (0.02) | 0.504 | 0.003 | 10 (0.02) | 10 (0.02) | 1.000 | 0.000 |
| Abatacept | 10 (0.02) | 13 (0.00) | 0.000 | 0.019 | 10 (0.02) | 0 (0.00) | 0.002 | 0.021 |
| Tofacitinib | 10 (0.02) | 157 (0.02) | 0.504 | 0.003 | 10 (0.02) | 10 (0.02) | 1.000 | 0.000 |
| Corticosteroids | 6728 (15.33) | 90524 (10.57) | 0.000 | 0.142 | 6725 (15.33) | 6506 (14.83) | 0.039 | 0.014 |
| Medical utilization |  |  |  |  |  |  |  |  |
| Ambulatory | 36082 (82.22) | 489467 (57.16) | 0.000 | 0.567 | 36079 (82.22) | 35822 (81.63) | 0.024 | 0.015 |
| Emergency | 6042 (13.77) | 129160 (15.09) | 0.000 | 0.037 | 6042 (13.77) | 5827 (13.28) | 0.034 | 0.014 |
| Inpatient Encounter | 1784 (4.07) | 47243 (5.52) | 0.000 | 0.068 | 1784 (4.07) | 1701 (3.88) | 0.151 | 0.010 |

**Table S11** Risk of outcomes exposed to Paxlovid compared to Non-Paxlovid in non-autoimmune population

|  | No. of event | |  |
| --- | --- | --- | --- |
|  | Paxlovid N = 43882 | Non-Paxlovid N = 43882 | HR |
| **Cerebrovascular complications** | 598 | 843 | **0.76 (0.68–0.84)** |
| Stroke | 516 | 754 | **0.73 (0.65–0.81)** |
| Transient ischemic attack | 138 | 172 | 0.88 (0.69–1.10) |
| **Arrhythmia** | 2084 | 2440 | **0.91 (0.85–0.96)** |
| Atrial fibrillation and flutter | 355 | 666 | **0.55 (0.48–0.62)** |
| Tachycardia | 978 | 1,027 | 1.03 (0.94–1.12) |
| Bradycardia | 611 | 689 | 0.97 (0.86–1.08) |
| Ventricular arrhythmia | 801 | 884 | 0.98 (0.88–1.07) |
| **Inflammatory heart disease** | 71 | 98 | 0.77 (0.56–1.04) |
| Pericarditis | 63 | 92 | 0.73 (0.52–1.00) |
| Myocarditis | 10 | 10 | 1.36 (0.46–3.93) |
| **Ischemic heart disease** | 321 | 434 | **0.80 (0.68–0.92)** |
| Acute coronary disease | 31 | 53 | 0.66 (0.41–1.03) |
| Myocardial infarction | 172 | 193 | 0.98 (0.79–1.21) |
| Ischemic cardiomyopathy | 35 | 63 | **0.60 (0.39–0.91)** |
| Angina | 135 | 193 | **0.75 (0.60–0.94)** |
| **Other cardiac disorders** | 411 | 664 | **0.65 (0.57–0.73)** |
| Heart failure | 317 | 542 | **0.62 (0.53–0.70)** |
| Non-ischemic cardiomyopathy | 125 | 181 | **0.72 (0.57–0.90)** |
| Cardiac arrest | 22 | 47 | **0.53 (0.31–0.88)** |
| Cardiogenic shock | 10 | 20 | **0.43 (0.18–0.98)** |
| **Thrombotic disorders** | 298 | 418 | **0.75 (0.64–0.86)** |
| Pulmonary embolism | 99 | 165 | **0.61 (0.47–0.78)** |
| Deep vein thrombosis | 150 | 210 | **0.77 (0.62–0.95)** |
| Superficial vein thrombosis | 102 | 120 | 0.90 (0.68–1.17) |
| **Major adverse cardiac events** | 1067 | 1498 | **0.75 (0.69–0.81)** |
| **Any cardiovascular outcome mentioned above** | 3094 | 3834 | **0.85 (0.80–0.88)** |
| **Mortality** | 191 | 572 | **0.34 (0.29–0.40)** |
| **Admission** | 4786 | 6482 | **0.74 (0.71–0.76)** |
| **ICU** | 259 | 535 | **0.51 (0.44–0.59)** |

If the patient's count is 10 or less, the results indicate a count of 10.

**Table S12** Sensitivity analysis for risk of outcomes exposed to Paxlovid compared to Non-Paxlovid indexed to COVID-19 Onset

|  | No. of event | |  |
| --- | --- | --- | --- |
|  | Paxlovid N = 9746 | Non-Paxlovid N = 9746 | HR |
| **Cerebrovascular complications** | 212 | 242 | 0.87 (0.72–1.05) |
| Stroke | 183 | 217 | 0.84 (0.68–1.02) |
| Transient ischemic attack | 50 | 59 | 0.84 (0.57–1.23) |
| **Arrhythmia** | 705 | 771 | **0.90 (0.81–0.99)** |
| Atrial fibrillation and flutter | 96 | 156 | **0.62 (0.48–0.80)** |
| Tachycardia | 380 | 376 | 0.99 (0.85–1.14) |
| Bradycardia | 202 | 211 | 0.96 (0.78–1.16) |
| Ventricular arrhythmia | 252 | 324 | **0.77 (0.65–0.91)** |
| **Inflammatory heart disease** | 35 | 50 | 0.71 (0.46–1.10) |
| Pericarditis | 32 | 49 | 0.66 (0.42–1.04) |
| Myocarditis | 10 | 10 | 1.52 (0.25–9.11) |
| **Ischemic heart disease** | 103 | 136 | **0.75 (0.57–0.97)** |
| Acute coronary disease | 10 | 18 | **0.34 (0.13–0.84)** |
| Myocardial infarction | 59 | 69 | 0.89 (0.62–1.26) |
| Ischemic cardiomyopathy | 10 | 23 | **0.45 (0.21–0.95)** |
| Angina | 40 | 52 | 0.75 (0.49–1.14) |
| **Other cardiac disorders** | 139 | 207 | **0.65 (0.52–0.80)** |
| Heart failure | 107 | 180 | **0.57 (0.44–0.72)** |
| Non-ischemic cardiomyopathy | 42 | 62 | **0.64 (0.42–0.95)** |
| Cardiac arrest | 10 | 10 | 1.49 (0.47–4.74) |
| Cardiogenic shock | 10 | 10 | 0.66 (0.21–2.02) |
| **Thrombotic disorders** | 95 | 124 | 0.77 (0.58–1.00) |
| Pulmonary embolism | 37 | 48 | 0.77 (0.49–1.18) |
| Deep vein thrombosis | 41 | 62 | **0.66 (0.43–0.98)** |
| Superficial vein thrombosis | 38 | 39 | 1.00 (0.63–1.57) |
| **Major adverse cardiac events** | 343 | 457 | **0.74 (0.63–0.84)** |
| **Any cardiovascular outcome mentioned above** | 1018 | 1149 | **0.87 (0.79–0.94)** |
| **Mortality** | 41 | 138 | **0.27 (0.18–0.39)** |
| **Admission** | 1494 | 1798 | **0.81 (0.76–0.87)** |
| **ICU** | 106 | 138 | 0.80 (0.61–1.02) |

If the patient's count is 10 or less, the results indicate a count of 10.

**Table S13** Sensitivity analysis for risk of outcomes exposed to Paxlovid compared to Non-Paxlovid with a five-day washout period

|  | No. of event/ N | |  |
| --- | --- | --- | --- |
|  | Paxlovid | Non-Paxlovid | HR |
| **Cerebrovascular complications** | 168/9461 | 192/9354 | 0.98 (0.79–1.20) |
| Stroke | 144/9461 | 167/9362 | 0.98 (0.78–1.23) |
| Transient ischemic attack | 40/9462 | 49/9421 | 0.93 (0.60–1.42) |
| **Arrhythmia** | 567/9451 | 589/9145 | 1.06 (0.93–1.18) |
| Atrial fibrillation and flutter | 76/9461 | 95/9383 | 0.90 (0.66–1.22) |
| Tachycardia | 302/9455 | 301/9294 | 1.11 (0.94–1.30) |
| Bradycardia | 168/9459 | 166/9312 | 1.17 (0.93–1.45) |
| Ventricular arrhythmia | 197/9462 | 241/9287 | 0.92 (0.76–1.11) |
| **Inflammatory heart disease** | 27/9462 | 36/9436 | 0.89 (0.53–1.47) |
| Pericarditis | 25/9462 | 34/9436 | 0.85 (0.50–1.43) |
| Myocarditis | 10/9462 | 10/9461 | 1.35 (0.19–9.48) |
| **Ischemic heart disease** | 75/9461 | 118/9360 | **0.73 (0.54–0.98)** |
| Acute coronary disease | 10/9462 | 13/9440 | 0.48 (0.18–1.25) |
| Myocardial infarction | 43/9461 | 58/9415 | 0.89 (0.59–1.33) |
| Ischemic cardiomyopathy | 10/9462 | 12/9446 | 0.54 (0.20–1.43) |
| Angina | 29/9462 | 58/9395 | **0.59 (0.37–0.92)** |
| **Other cardiac disorders** | 100/9460 | 150/9367 | **0.73 (0.56–0.94)** |
| Heart failure | 81/9461 | 127/9377 | **0.71 (0.53–0.93)** |
| Non-ischemic cardiomyopathy | 28/9461 | 37/9425 | 0.82 (0.49–1.33) |
| Cardiac arrest | 10/9462 | 10/9456 | 1.01 (0.25–4.01) |
| Cardiogenic shock | 10/9462 | 10/9457 | 0.82 (0.18–3.66) |
| **Thrombotic disorders** | 70/9462 | 97/9394 | 0.79 (0.57–1.07) |
| Pulmonary embolism | 27/9462 | 34/9439 | 0.82 (0.49–1.36) |
| Deep vein thrombosis | 26/9462 | 39/9425 | 0.72 (0.43–1.18) |
| Superficial vein thrombosis | 28/9462 | 43/9429 | 0.74 (0.45–1.20) |
| **Major adverse cardiac events** | 263/9460 | 355/9270 | **0.83 (0.70–0.97)** |
| **Any cardiovascular outcome mentioned above** | 820/9448 | 900/9052 | 0.99 (0.89–1.08) |
| **Mortality** | 35/9395 | 118/9341 | **0.31 (0.21–0.45)** |
| **Admission** | 534/6508 | 652/6114 | **0.86 (0.76–0.96)** |
| **ICU** | 70/9368 | 116/9260 | **0.67 (0.49–0.90)** |

If the patient's count is 10 or less, the results indicate a count of 10.


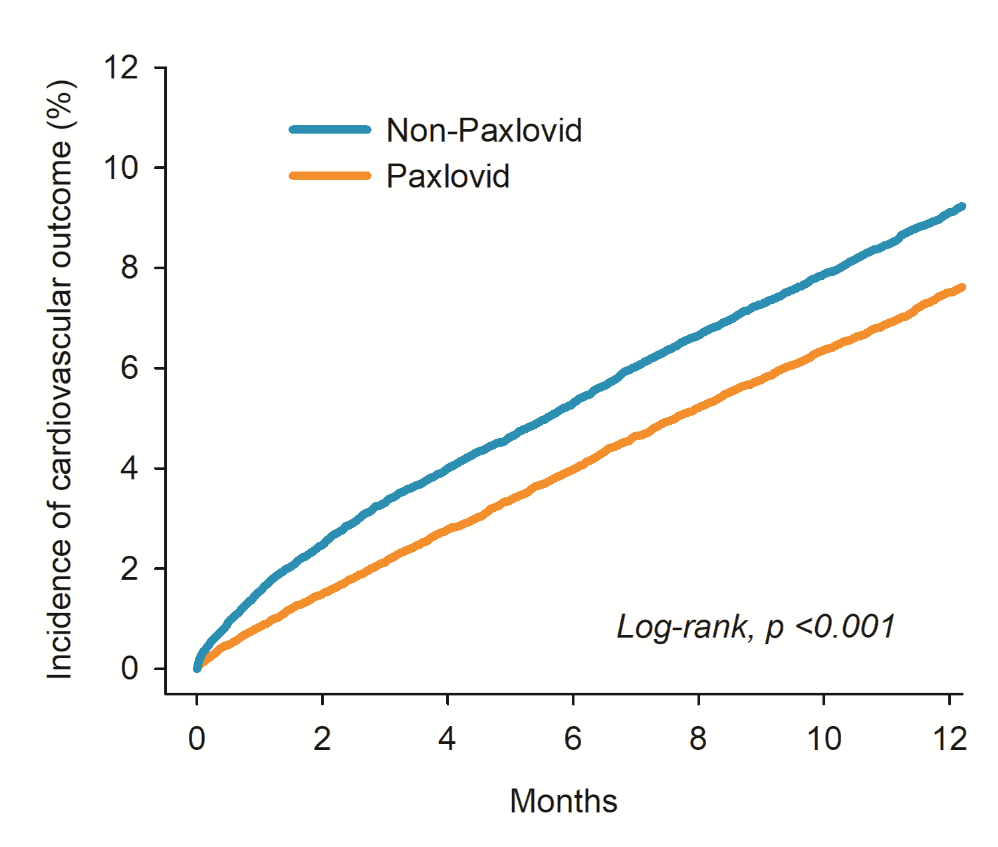


**FigS1** Kaplan-Meier plot for risk of cardiovascular diseases in non-autoimmune population
