# Supplementary material for: Paxlovid use is associated with lower risk of cardiovascular diseases in COVID-19 patients with autoimmune rheumatic diseases: a retrospective cohort study
Source: BMC Med. 2024 Mar 13;22:117. doi: 10.1186/s12916-024-03331-0 (PMC10938827; doi:10.1186/s12916-024-03331-0)
Supplement: Supplementary file 2 — Additional file 2: Table S1. STROBE Statement—Checklist of items that should be included in reports of cohort studies. [file 12916_2024_3331_MOESM2_ESM.docx]

**Table S1**: STROBE Statement—Checklist of items that should be included in reports of ***cohort studies***

|  | Item No | Recommendation | Page No | Description |
| --- | --- | --- | --- | --- |
| **Title and abstract** | 1 | (*a*) Indicate the study’s design with a commonly used term in the title or the abstract | 1 | a retrospective cohort study |
|  |  | (*b*) Provide in the abstract an informative and balanced summary of what was done and what was found. | 2-3 | Methods and Conclusion part |
| Introduction | | | |  |
| Background/rationale | 2 | Explain the scientific background and rationale for the investigation being reported | 4 | COVID-19, Paxlovid, AIRD |
| Objectives | 3 | State specific objectives, including any prespecified hypotheses | 4 | to provide some evidence for Paxlovid use in  COVID-19 patients with AIRD |
| Methods | | | |  |
| Study design | 4 | Present key elements of study design early in the paper | 5 | US Collaborative Network in the TriNetX |
| Setting | 5 | Describe the setting, locations, and relevant dates, including periods of recruitment, exposure, follow-up, and data collection | 4-5 | the US Collaborative Network in TriNetX was  used to build a cohort out of the more than 92  million participants.（Methods section）  Lines 92-100 |
| Participants | 6 | (*a*) Give the eligibility criteria, and the sources and methods of selection of participants. Describe methods of follow-up | 6 | A total of 5,671,395 patients with AIRD were  enrolled between January1, 2010 and December  31, 2021 from 92,985,898 participants in US  Collaborative Network.  Age ≥18 y/o and diagnosis of COVID-19 (test  positive or ICD-10-CM = U07.1) (2022/1/1~2022/12/31)  N = 238142  Exclusion   1. Used molnupiravir, remdesivir after diagnosis   of COVID-19, N = 269   1. Diagnosed cardiovascular diseases before   index date, N = 7322  12 months follow up |
|  |  | (*b*) For matched studies, give matching criteria and number of exposed and unexposed | 6 | Paxlovid group and Non-Paxlovid group  （Methods section, Participants part）  Lines 102-119 |
| Variables | 7 | Clearly define all outcomes, exposures, predictors, potential confounders, and effect modifiers. Give diagnostic criteria, if applicable | 7-8 | AIRDs; social economic status; comorbidities,  medications, and medical utilization  outcomes: cardiovascular complications and  severe conditions  Lines 125-158 |
| Data sources/ measurement | 8* | For each variable of interest, give sources of data and details of methods of assessment (measurement). Describe comparability of assessment methods if there is more than one group | 7-8 | ICD10, ICD-CM.  US Collaborative Network in TriNetX |
| Bias | 9 | Describe any efforts to address potential sources of bias | 8 | propensity score matching  lines 141-151  five-day washout period supplementary Table 5 |
| Study size | 10 | Explain how the study size was arrived at | 5-6 | After PSM, 8,803 participants in the Paxlovid  and 8,803 comparisons in the Non-Paxlovid  groups were selected.  （Participants part, Lines 103-119） |
| Quantitative variables | 11 | Explain how quantitative variables were handled in the analyses. If applicable, describe which groupings were chosen and why | 8 | 95% confidence interval (95% CI) |
| Statistical methods | 12 | (*a*) Describe all statistical methods, including those used to control for confounding | 8-9 | Kaplan-Meier analysis, Cox proportional hazard  Model, HR |
|  |  | (*b*) Describe any methods used to examine subgroups and interactions |  | with respect to sex, age, race and autoimmune diseases groups. |
|  |  | (*c*) Explain how missing data were addressed |  | possible differences between the early and late  use of Paxlovid  within the first day of COVID-19 and 2-5days |
|  |  | (*d*) If applicable, explain how loss to follow-up was addressed |  | Statical analyses part, lines 160-178 |
|  |  | (*e*) Describe any sensitivity analyses |  |  |
| Results | | |  |  |
| Participants | 13* | (a) Report numbers of individuals at each stage of study—eg numbers potentially eligible, examined for eligibility, confirmed eligible, included in the study, completing follow-up, and analysed |  |  |
|  |  | (b) Give reasons for non-participation at each stage | 7 | Figure 1 Flow chart of cohort construction |
|  |  | (c) Consider use of a flow diagram |  |  |
| Descriptive data | 14* | (a) Give characteristics of study participants (eg demographic, clinical, social) and information on exposures and potential confounders | 10 | Baseline characteristics of the participants part  Table1  (Lines 180-192) |
|  |  | (b) Indicate number of participants with missing data for each variable of interest | 10 |  |
|  |  | (c) Summarise follow-up time (eg, average and total amount) | 11 |  |
| Outcome data | 15* | Report numbers of outcome events or summary measures over time | 11-13 | Cardiovascular outcomes  Figure2, Figure3, and Table S2  Lines 193-221 |

| Main results | 16 | (*a*) Give unadjusted estimates and, if applicable, confounder-adjusted estimates and their precision (eg, 95% confidence interval). Make clear which confounders were adjusted for and why they were included | 11 | Figure2 and Table S2 |
| --- | --- | --- | --- | --- |
|  |  | (*b*) Report category boundaries when continuous variables were categorized | 11 |  |
|  |  | (*c*) If relevant, consider translating estimates of relative risk into absolute risk for a meaningful time period | NA |  |
| na | 17 | Report other analyses done—eg analyses of subgroups and interactions, and sensitivity analyses | 12-16 | subgroups were evaluated based on sex, age and  race, different autoimmune diseases.  Figure4,5 Table2, Table S2-S7  Lines 223-300 |
| Discussion | | | |  |
| Key results | 18 | Summarise key results with reference to study objectives | 17 | treatment with Paxlovid, particularly within the  first day of COVID-19 diagnosis could  significantly reduce the risks of cardiovascular  complications in COVID-19-surviving patients  with AIRD |
| Limitations | 19 | Discuss limitations of the study, taking into account sources of potential bias or imprecision. Discuss both direction and magnitude of any potential bias | 19 | Lines 368-389  the information of disease activity of the  autoimmune diseases; Paxlovid dose data;  limitations of the TrinetX platform;  potential for immortal time bias; a racial bias;  healthcare insurance; drug-drug interaction |
| Interpretation | 20 | Give a cautious overall interpretation of results considering objectives, limitations, multiplicity of analyses, results from similar studies, and other relevant evidence | 17-19 | Discussion part. Lines 302-389 |
| Generalisability | 21 | Discuss the generalisability (external validity) of the study results | 19 | The number of Asian people in our study was  rather small, which may have produced a racial  bias in the results. |
| Other information | | | |  |
| Funding | 22 | Give the source of funding and the role of the funders for the present study and, if applicable, for the original study on which the present article is based | 20-21 | Funding part |

*Give information separately for exposed and unexposed groups.

**Note:** An Explanation and Elaboration article discusses each checklist item and gives methodological background and published examples of transparent reporting. The STROBE checklist is best used in conjunction with this article (freely available on the Web sites of PLoS Medicine at http://www.plosmedicine.org/, Annals of Internal Medicine at http://www.annals.org/, and Epidemiology at http://www.epidem.com/). Information on the STROBE Initiative is available at http://www.strobe-statement.org.
